# Job crafting and employees’ general health: the role of work–nonwork facilitation and perceived boundary control

**DOI:** 10.1186/s12889-022-13569-z

**Published:** 2022-06-15

**Authors:** Yanwei Shi, Dan Li, Nan Zhang, Ping Jiang, Deng Yuling, Julan Xie, Jun Yang

**Affiliations:** 1 Department of Human Resource Management, Shanghai Normal University, Shanghai, 200234 China; 2 Hainan key novel thinktank “Hainan Medical University ‘One Health’ Research Center, Hainan Medical University, Haikou, 570216 China; 3 Beijing Key Laboratory of Applied Experimental Psychology, National Demonstration Center for Experimental Psychology Education (Beijing Normal University), Faculty of Psychology, Beijing Normal University, 19 Xinjiekouwai St., HaiDian District, Beijing, 100875 China; 4 Department of Nursing, Shanghai Pudong New area People’s Hospital, Shanghai, 201299 China; 5 Health Management Center, the Third Xiangya Hospital, Central South University, Changsha, 410083 China; 6 School of Business, Central South University, Changsha, 410083 China

**Keywords:** Conservation of resources theory, General health, Job crafting, Perceived boundary control, Work–nonwork facilitation

## Abstract

**Background:**

Job crafting is associated with positive work–related outcomes, but its effects on nonwork–related outcomes are unclear. The conservation of resources theory informed the hypotheses that work–nonwork facilitation mediates the relationship between job crafting and general health, and this mediation process is moderated by perceived boundary control.

**Methods:**

Using a two–wave design, 383 employees from a range of work settings completed questionnaires in which they rated job crafting, work–nonwork facilitation, general health and perceived boundary control.

**Results:**

Moderated mediation analysis showed that work–nonwork facilitation mediated the relationship between job crafting and employee general health. Further, perceived boundary control moderated this indirect effect, such that the indirect effect was stronger for employees with high perceived boundary control than those with low perceived boundary control.

**Conclusions:**

This study is an important step forward in understanding the effect of job crafting on nonwork domains, and in clarifying “how” and “when” job crafting might affect employees’ general health. Further, the results have practical implications for fostering employee general health.

**Supplementary Information:**

The online version contains supplementary material available at 10.1186/s12889-022-13569-z.

## Background

In times of rapid organizational change, employees often need to take initiative to change the conditions of their existing jobs [1, 2]. Job crafting, which refers to self–initiated changes in one’s job or workplace, has received increasing attention from researchers and practitioners [3, 4]. Job crafting involves changes that employees make in their job demands and job resources to attain and/or optimize their personal or work goals, such as seeking social support and starting new projects [2]. Tims et al. suggested that it has four dimensions: increasing structural job resources, increasing social job resources, increasing challenging job demands, and decreasing hindering job demands [2]. Job crafting appears to have a positive effect on various work–related attitudes and behaviors, such as improved job performance [5, 6], increased work engagement [6, 7], work meaningfulness [8], and job satisfaction [9, 10].

However, compared to the extensive literature on the effect of job crafting on work–related outcomes [6, 8, 11], there has been little research on the potential effects of job crafting on employees’ nonwork lives. Indirect evidence of this link comes from a study showing that employees’ general job behaviors can spillover to impact behaviors, thoughts and feelings in the nonwork domain [12]. Accordingly, job crafting may also spillover to the nonwork domain to influence employees’ nonwork lives.

Recent research has focused on the link between job crafting and good health as a nonwork outcome [13–15]. However, only one of these studies [14] focused on the effect of job crafting on general health. General health reflects the individual’s perceptions of physical symptoms, anxiety symptoms, sleep disturbance, social functioning and depression symptoms [16, 17], which reflects positive and negative aspects of health [18]. Further, previous study suggests that general health reflects mental health as much as physical health [18]. Given that the general health is a broader outcome than mental and physical health, compared to explore the effect of job crafting on mental or physical health, it is important to test the relationship between job crafting and general health.

However, it remains unclear how the effect of job crafting on general health unfolds. Given that general health has important implications for employees’ lives [19, 20] and the organization’s productivity [21, 22], it would be useful to understand why job crafting has these positive effects. It would also be useful to determine if these positive effects are stronger for some employees than others. Addressing these questions can facilitate our understanding of how to better promote employees’ general health. Thus, the current study tested potential mediation process and moderating factors that could elucidate the nature of this relationship.

Evidence of mediation begins with evidence of a direct link between job crafting and non–domain outcomes. The Conservation of Resources (COR) theory provides a helpful framework for forming hypotheses about this link. Specifically, COR theory proposes that individuals may invest their current resources into building new resources, consequently sustaining and protecting their well–being [23]. Further, Wayne et al. [24] proposed that the resources generated by work can potentially positively spillover into the nonwork domain, where they can be applied and reinforced.

However, previous studies overlooked the mediators of the association between job crafting and general health. We argue that employees can use job crafting to optimize their job resources (e.g., developmental opportunities and job control); these resources can further spillover to and promote employees’ functioning in nonwork domains [24] via enhancing work–nonwork facilitation. Further, a high level of work–nonwork facilitation enables employees to apply, sustain, and reinforce the gains in nonwork domains to improve general health. In the current study, we focus on work–nonwork facilitation as a mediator in the relationship between job crafting and general health.

Similarly, boundary conditions of the mediation process underlying between job crafting and general health have been rarely examined thus far. Because job crafting appears to be an effective way to improve the fit between the job and the worker, we need to understand how personal resources affect the indirect effect of job crafting on employee outcomes. Personal resources (e.g., key skills and personal traits) play an important role in the management of job–related resources [23–25].

One personal resource is thought to be perceived boundary control, which is the perception that one “can control the timing, frequency, and direction” of mental, physical, and temporal transitions between the work and family domains [26]. The results of one study suggested that perceived boundary control moderates the relationship between job resources and work–family facilitation [27]. The concept of resource enhancement [28] is that resources that are generated from job crafting have a greater impact on work–nonwork facilitation and general health for individuals with more personal resources (e.g., high perceived boundary control). This greater impact occurs because the effects of multiple resources are complementary and synergistic [28]. We argue that perceived boundary control moderates the association between job crafting and employees’ general health.

**Fig. 1:**
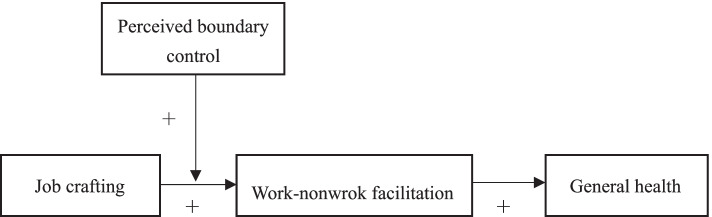
Hypothesized moderated mediation model linking lack of job crafting to employee general health through work-nonwork facilitation

Our study fills these gaps in the literature by testing work–nonwork facilitation as a mechanism by which job crafting enhances general health, and by taking into account individual differences in perceived boundary control. We tested this conceptual model using tests of moderated mediation. Figure 1 shows the conceptual model.

Our research makes two contributions to the literature. First, integrating the COR theory and research on job crafting contributes to a deeper understanding of the relationship between job crafting and employee general health. This integration provided the framework for examining work–nonwork facilitation as a potential mediator of this relationship. Second, the present study tests an individual difference variable, namely perceived boundary control, as a moderator of this mediation process. To our knowledge, this is the first study to examine a boundary condition of indirect effect of job crafting on general health, which extends our understanding of when job crafting is more beneficial.

## Theoretical background

### Conservation of resources (COR) theory

COR theory provides the theoretical foundation for exploring how job crafting may promote work–nonwork facilitation, which in turn may increase employee general health. COR theory proposes that individuals may invest their current resources into building new resources, consequently sustaining and protecting their well–being [23]. Further, person–related resources play an important role in the management of other–related resources [23].

Based on COR theory, employees invest the resources generated from job crafting to gain new resources, which can spillover to the nonwork domain, fostering work–nonwork facilitation. In turn, higher work–nonwork facilitation can enable employees to apply, sustain, and reinforce resources in the nonwork–domains to improve general health. Further, employees with higher person–related resources (perceived boundary control) can better benefit from job crafting. In the following sections, we will elaborate on these arguments and propose specific hypotheses.

### Hypotheses

#### Job crafting and work–nonwork facilitation

The resources gained from job crafting in the work domain may affect the quality of life in nonwork domains [29]. Employees who proactively craft their jobs will be able to better shape job demands and resources to fit their needs and abilities [2] and hence will be better equipped with resources to fulfill their work obligations. These benefits could influence the work–nonwork facilitation.

Work–nonwork facilitation refers to how resources gained at work promote functioning or positive effects during time devoted to family or personal interests [24, 30]. Work–nonwork research predominantly has focused on two pivotal characteristics of the work–nonwork facilitation: work–family facilitation and work–self facilitation [31]. Specifically, work–family facilitation refers the extent to which an individual’s engagement in the work domain provides gains that enhance functioning in the family domain [24]. Work–self facilitation refers to the extent to which resources gained at work promote functioning or positive affect during time devoted to personal interests [30]. The “self” is all of the person’s qualities that make him or her unique, including preferences, interests, hobbies and wishes, and is independent of work and family roles [30].

Employees who have extensive job resources or fewer job demands will have sufficient resources to deal with demands in their nonwork domain, promoting work–nonwork facilitation [32]. Accordingly, if employees proactively craft their job resources and job demands, they have sufficient resources in their work, and can use these resources to improve the quality of life outside of work.

COR theory provides a theoretical basis for the above view. In line with the idea of COR theory [23], job crafting can increase resources such as positive emotions [33], self–efficacy [34], and meaningfulness [8], and these additional new resources facilitate functioning in nonwork roles. In other words, we argue that resources resulting from job crafting generate even more resources, which are then transmitted to nonwork domains through a process of positive psychological spillover. In addition, given that the resources gained from the work domain can be applied, sustained, and reinforced in nonwork domains [24], the resources generated from job crafting create a greater potential for more work–nonwork facilitation.

Previous research has provided initial evidence that supports the above view. For example, researchers found that job crafting had a positive relationship with employee work–family enrichment [35, 36]. Similarly, Tresi and Mihelič [34] found that job crafting positively affected work–self facilitation. Taken together, these studies suggest that the resources produced by job crafting can promote work–nonwork facilitation. Thus, we proposed the following:

##### Hypothesis 1

Job crafting will positively predict work–nonwork facilitation.

#### Work–nonwork facilitation and general health

It appears that high work–family facilitation promotes health [37]. Further, the resources that promote work–nonwork facilitation can lead to positive outcomes, including better health [24]. For example, individuals who experience greater work–nonwork facilitation are more likely to report higher job, family, and life satisfaction [38, 39] and better mental health [40]. Thus, we argue that the resources associated with work–nonwork facilitation enhance performance in nonwork domains, thus increasing general health.

The COR theory [23] provides a theoretical explanation for the relationship between work–nonwork facilitation and employee general health. The theory argues that those who have greater resources will be less vulnerable to resource loss. Accordingly, employees with high work–nonwork facilitation will be more capable of solving problems in stressful situations, resulting in better general health. Based on theoretical grounds and empirical studies, we expected the following:

##### Hypothesis 2

Work–nonwork facilitation will positively predict employee general health.

#### The mediating role of work–nonwork facilitation

Employees tend to maximize resources from job crafting to obtain other resources that can promote work–nonwork facilitation [24]. In turn, a high level of work–nonwork facilitation enables employees to apply, sustain, and reinforce the gains that derive from job crafting in nonwork domains to improve health. Therefore, we propose that work–nonwork facilitation mediates the effect of job crafting on general health.

COR theory proposes that individuals may invest their current resources in building new resources and consequently in sustaining and protecting their well–being [23]. According to this theory, employees invest their resources generated by job crafting into gaining new resources that spill over to nonwork domains (work–nonwork facilitation), ultimately promoting general health. Therefore, we propose that job crafting is a way for employees to gain resources that promote work–nonwork facilitation, and job crafting helps them to accumulate further resources to maintain general health.

Although work–nonwork facilitation has not been tested as a mediator of the relationship between job crafting and general health, previous findings provide initial support for the idea. For example, work–nonwork facilitation has been found to mediate the relationship between family–supportive supervisor behaviors and well–being [28, 41]. Given that job crafting is a job resource and general health is an indicator of wellbeing, we argue that work–nonwork facilitation may also mediate the effect of job crafting on general health. Based on the theoretical considerations and previous research, we hypothesized:

##### Hypothesis 3

Work–nonwork facilitation will mediate the relationship between job crafting and employee general health.

#### The moderating role of perceived boundary control

Perceived boundary control is the psychological interpretation of perceived control over one’s boundary environment and considered an important personal psychological resource [26, 28]. Individuals with higher perceived boundary control believe they can control the timing, frequency, and direction of boundary crossings to fit their identities and multiple role demands [26]. Previous studies found that people with high boundary control also perceived themselves as having additional resources (e.g., psychological job control, self–identity) [26]. In our conceptual model, we tested whether perceived boundary control, as an important personal resource [42], is a moderator in the first link of the mediation process.

Our proposal—that perceived boundary control will strengthen the association between job crafting and work–nonwork facilitation—fits well with the concept of resource enhancement [28]. This term refers to the idea that the availability of a resource has a greater impact for individuals who have access to other resources, because of the complementary and synergistic effects of multiple resources [24]. Based on the concept of resource enhancement, we would predict that individuals with higher perceived boundary control are more likely to benefit from job crafting to promote work–nonwork facilitation. However, individuals in resource depletion are more likely to withdraw their efforts at acquiring more resources [43]. Accordingly, low perceived boundary control may make it difficult for employees to manage the resources generated by job crafting (e.g., positive emotion, well–being) that help them balance work and nonwork.

There is some initial evidence that supports the above view. For example, Jiang et al. [27] found that the positive effect of a family–supportive supervisor on work–family enrichment was stronger for employees who had higher perceived boundary control than those who had lower perceived boundary control. Similarly, high perceived boundary control was shown to strengthen the relationship between family–supportive supervisor behaviors and work engagement [44]. Based on the COR theory and empirical studies, we proposed the following moderation hypothesis:

##### Hypothesis 4

Perceived boundary control will moderate the relationship between job crafting and employee work–nonwork facilitation, such that the relationship will be stronger for employees with high perceived boundary control than for those with low perceived boundary control.

### The mediating role of work–nonwork facilitation and the moderating role of perceived boundary control

According to Muller, Judd and Yzerbyt’s [45] suggestions, we tested a moderated mediation model that combined the aforementioned mediation and moderation hypotheses. In this model, employees who have high perceived boundary control are able to obtain more resources from job crafting and to more effectively use those resources to promote work–nonwork facilitation, resulting in better general health.

#### Hypothesis 5

Perceived boundary control will moderate the indirect effect of job crafting on general health through work–nonwork facilitation, such that the indirect effect will be stronger for employees with high perceived boundary control than those with low perceived boundary control.

## Method

### Participants and procedure

We recruited participants who worked for different companies in central and eastern China. We used a snowball approach to recruit participants, all of whom were employed full–time. After recruiting an initial group of employees, we then invited the employees’ friends to participate, and then their friends and family.

Similar to previous study [46], we collected data twice with a 3–month interval between the two waves. After participants provided informed consent, research assistants used WeChat to send a link to a web–based set of questionnaires. The questionnaires asked about job crafting, perceived boundary control and the control variables. At Time 1, 520 employees were invited to participate, and 459 provided valid data, resulting in a response rate of 88.27%. Sixty-one participants were removed from the final analysis because they did not complete any part of the questionnaires (30 participants) or had more than 30% missing data (31 participants).

At Time 2, research assistants again used WeChat to send a link to the 459 participants who completed the questionnaires at Time 1. This web–based questionnaire only asked about work–nonwork facilitation and general health. A total of 383 valid questionnaires were received (83.44% response rate). The final sample of 383 employees had a mean age of 29.94 (SD = 5.67) and worked 43.72 (SD = 8.58) hours a week on average. Of these participants, 239 (62.40%) were female, 150 (39.16%) had at least one child under the age of 18, and 158 (41.25%) had one or more elderly persons to take care of.

The present study received the university’s research ethics committee’s approval. The anonymity of participants’ responses was guaranteed. Participants were asked to provide the last four digits of their thirteen–digit phone number in order to match the questionnaire data from Time 1 and Time 2. Participants who completed the survey at both time points were given 20 RMB, about 3 US Dollars, to thank them for their time and effort.

### Measures

#### Job crafting (T_1_)

The Job Crafting Questionnaire was used to measure the extent to which the employees make changes in their job demands and job resources [2]. The measure includes 21 items on four subscales: increasing structural job resources (5 items; e.g., “I try to learn new things at work”), increasing social job resources (5 items; e.g., “I ask colleagues for advice”), increasing challenging job demands (5 items; e.g., “I ask for more responsibilities”), and decreasing hindering job demands (6 items; e.g., “I try to ensure that my work is less physically intense”). The items were rated on a 5–point Likert scale (1 = strongly disagree, 5 = strongly agree), with high scores indicating high job crafting behavior. In the current study, the Cronbach’s alpha of the scale was 0.86.

#### Work–nonwork facilitation (T2)

Referencing previous research [31], the construct of work–nonwork facilitation represented a combination of work–family facilitation and work–self facilitation. Specifically, the work–family facilitation scale [47] and work–self facilitation scale [48] were combined to assess work–nonwork facilitation. Participants responded to statements on a 5–point Likert scale (1 = never, 5 = always). Example items include, “Having a good day on your job makes you a better companion when you get home” (work–family facilitation), and “After work you really feel like pursuing your personal interests” (work–self facilitation). The responses were averaged across the items from the work–family facilitation scale (4 items) and the work–self facilitation scale (4 items), with higher scores indicating higher work–nonwork facilitation. In the current study, the Cronbach’s alphas of the work–nonwork facilitation, work–family facilitation and work–self facilitation scales were 0.81, 0.79 and 0.77 respectively.

#### General health (T_2_)

We measured employees’ general health with the 12–item General Health Questionnaire [49]. This questionnaire contains 12 items that are rated on a 4–point Likert scale (1 = never, 4 = always). Example items are “I am able to concentrate” and “I lose sleep because of worry (reverse scored).” Half of the items are reverse scored so that a higher score reflects a higher level of general health. The Cronbach’s alpha of the scale was 0.87 in the current study.

#### Perceived boundary control (T_1_)

We used the 4-item perceived boundary control scale by Kossek et al. [26] to measure the extent to which employee perceived control over their boundary environment. An example item is, “I control whether I am able to keep my work and personal life separate.” The items were rated on a 5-point Likert scale (1 = strongly disagree, 5 = strongly agree), with higher scores indicating higher perceived boundary control. In the current study, the Cronbach’s alpha of the scale was 0.87.

#### Control variables (T_1_)

General health has been found to be higher among women and negatively correlated with age [18, 50]. Thus, we chose gender (1 = male; 0 = female) and age as control variables. Further, taking care of children or elderly persons has been shown to be stressful, and stress is related to worse general health [51]. We thus included whether participants had children under the age of 18 (1 = yes; 0 = no), and whether they needed to provide care for one or more elderly persons (1 = yes; 0 = no) as control variables.

### Data analysis

To test our hypotheses, we used the SPSS macro PROCESS [52], which tests complex models that include both mediator and moderator variables. Specifically, we used PROCESS Model 4 to test the mediating effect of work–nonwork facilitation in the relationship between job crafting and general health (Hypotheses 1, 2, and 3). Additionally, we used PROCESS Model 7 to test the moderated mediation effect (Hypotheses 4 and 5). Finally, we used PROCESS Model 1 to produce the output used to probe and graph significant interactions. In the present study, bootstrapped bias-corrected 95% confidence intervals (CI) for the indirect effects were generated using 5000 iterations of bootstrapping. A result is considered significant when the 95% confidence interval does not include 0.

## Results

### Descriptive statistics

Table 1 shows the means, standard deviations, and correlations among all of the variables. Results showed that job crafting was positively related to general health (*r* = 0.28, *p* < 0.001) and work–nonwork facilitation (*r* = 0.32, *p* < 0.001). Additionally, work–nonwork facilitation was positively related to general health (*r* = 0.32, *p* < 0.001).

**Table 1:** Means, Standard Deviations, and Correlations Among Study Variables

Variables	*M*	*SD*	1	2	3	4	5	6	7	8
1.Gender	–	–	–							
2.Age	29.94	5.57	0.01	–						
3.Children	–	–	−0.01	−0.46^***^	–					
4.Elder care	–	–	0.06	−0.38^***^	0.43^***^	–				
5.Job crafting (T_1_)	3.57	0.52	−0.04	0.03	−0.07	− 0.07	–			
6.Perceived boundary control (T_1_)	3.46	0.76	−0.02	0.02	0.03	0.09	0.26^***^	–		
7.Work-nonwork facilitation (T_2_)	3.52	0.62	−0.02	0.01	0.01	−0.06	0.32^***^	0.46^***^	–	
8.General health (T_2_)	3.04	0.45	0.07	0.10^*^	−0.02	0.10^*^	0.28^***^	0.33^***^	0.32^***^	–

T_1_ represents the first measurement, and T_2_ represents the second measurement; Children = children under 18 years of age; Elder care = whether there are Elder man to take care;^*^p < 0.05, ^**^p < 0.01, ^***^ *p <* 0.001, similarly hereinafter

### Hypothesis testing

#### Mediated effects

As Table 2 (Eq. 1) shows, job crafting positively predicted work–nonwork facilitation (*B* = 0.39, *SE* = 0.06, *p* < 0.001), supporting Hypothesis 1. As Table 2 (Eq. 3) shows, work–nonwork facilitation positively predicted general health (*B* = 0.19, *SE* = 0.04, *p* < 0.001), supporting Hypothesis 2. The mediating effect of work–nonwork facilitation in the relationship between job crafting and general health was significant (indirect effect = 0.07, *SE* = 0.21, 95% CI [0.04, 0.12]), supporting Hypothesis 3.

**Table 2:** Regression results for meditation effect of work-nonwork facilitation and moderated meditation effect

Predictors	Work-nonwork facilitation (T_2_)				General health (T_2_)	
	Equation 1		Equation 2		Equation 3	
	*B*	*SE*	*B*	*SE*	*B*	*SE*
Gender	0.00	0.06	0.02	0.06	0.06	0.04
Age	0.00	0.01	0.00	0.01	0.01	0.01
Children	0.05	0.07	0.03	0.07	−0.02	0.05
Elder care	−0.07	0.07	−0.12	0.06	0.18^***^	0.05
Job crafting (T_1_)	0.39^***^	0.06	0.28^***^	0.05	0.17^***^	0.04
Work-nonwork facilitation (T_2_)					0.19^***^	0.04
Perceived boundary control (T_1_)			0.33^***^	0.04		
Job crafting × Perceived boundary control			0.23^***^	0.06		
* R*^*2*^	0.11		0.29		0.18	
* F*	9.03^***^		21.68^***^		13.89^***^	

T_1_ represents the first measurement, and T_2_ represents the second measurement; ^*^ *p <* 0.05, ^**^ *p <* 0.01, ^***^ *p <* 0.001

#### Moderated mediation effects

**Fig. 2:**
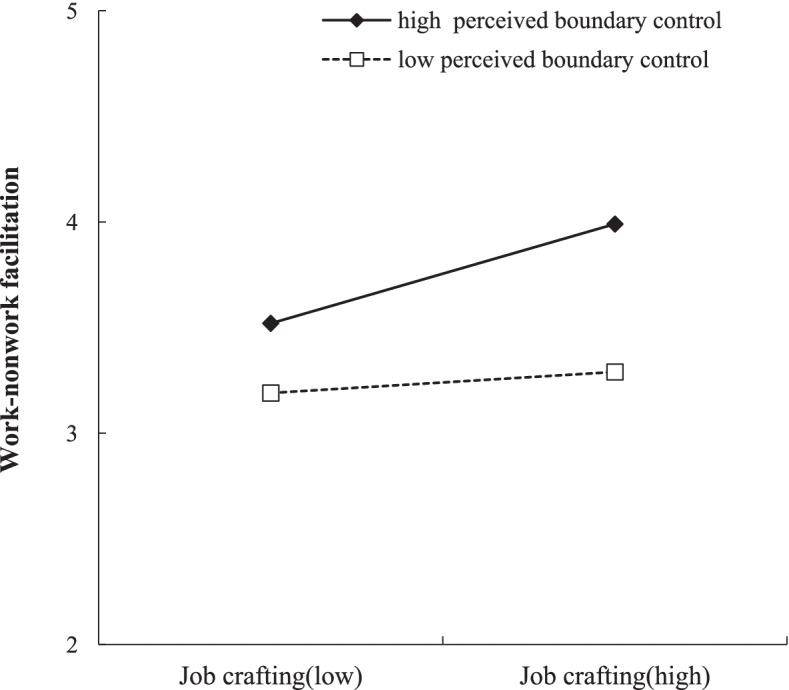
Perceived boundary control moderates the relation between job crafting and work-nonwork facilitation

We tested the moderation effect of perceived boundary control on the first link of the mediation process, namely the relationship between job crafting and work–nonwork facilitation (Hypothesis 4). As shown in Table 2 (Eq. 2), the interaction between job crafting and perceived boundary control positively predicted work–nonwork facilitation (*B* = 0.23, *SE* = 0.06, *p* < 0.001). Further, simple slope analysis showed that the relationship between job crafting and work–nonwork facilitation was significantly more positive for employees with high perceived boundary control (1SD above the mean, *B*_simple_ = 0.46, *p* < 0.001, 95% CI [0.30, 0.61]) than for those with low perceived boundary control (1SD below the mean, *B*_simple_ = 0.10, *p* = 0.14, 95% CI [− 0.04, 0.24]). Thus, the results supported Hypothesis 4. Figure 2 shows the interaction plot.

Hypothesis 5 predicted that perceived boundary control would moderate the mediating effect of work–nonwork facilitation in the relationship between job crafting and general health. Results in Table 3 showed that the indirect effect of job crafting on general health through work–nonwork facilitation was significantly stronger for employees with high perceived boundary control (*B* = 0.09, boot *SE* = 0.03, 95% CI [0.04, 0.14]) than those with low perceived boundary control (*B* = 0.02, boot *SE* = 0.02, 95% CI [− 0.02, 0.06]), and the index of moderated mediation was significant (index = 0.04; boot *SE* = 0.02, 95% CI [0.01, 0.09]). Thus, the results supported Hypothesis 5.

**Table 3:** Conditional indirect effects of job crafting on general health at different values of perceived boundary control

	Perceived boundary control	Effect	SE_(boot)_	95%*CI*
	−0.76	0.02	0.02	[−0.02, 0.06]
Work-nonwork facilitation	0	0.05	0.02	[0.03, 0.09]
	0.76	0.09	0.03	[0.04, 0.14]

#### Additional analyses

The results of the main analyses supported the hypothesis that job crafting was associated with general health via work–nonwork facilitation. They also supported the hypothesis that this mediation process would be moderated by boundary control. In additional analyses, we examine whether these effects would also be found when each of the four dimensions of job crafting (rather than the overall job crafting score) was entered as the independent variable.

The first set of additional analyses concerned the indirect effects of the different dimensions of job crafting on general health via work–nonwork facilitation. Specifically, for increasing job resources, indirect effect = 0.06, 95% CI [0.03, 0.09]; for increasing challenging job demands, indirect effect = 0.05, 95% CI [0.03, 0.08]; and for decreasing hindering job demands, indirect effect = 0.06, 95% CI [0.03, 0.10]. These results suggest that the mediation process was similar when the overall job crafting score was the independent variable and when each dimension of job crafting was the independent variable.

The second set of additional analyses concerned the index of moderated mediation. There was evidence of moderated mediation when the independent variable was increasing job resources, index = 0.038, boot *SE* = 0.019, 95% CI [0.007, 0.084]; and when the independent variable was increasing challenging job demands, index = 0.028, boot *SE* = 0.013, 95% CI [0.007, 0.057]. However, perceived boundary control did not moderate the mediation process when decreasing hindering demand was the independent variable (index = 0.012, boot *SE* = 0.016, 95% CI [− 0.019, 0.043]). According to earlier research, employees who decrease hindering job demands via job crafting are at lower risk of resources depletion (such as low burnout, exhaustion) [6], rather than increasing resources. Thus, individuals with high perceived boundary control are less likely to benefit from decreasing hindering job demands to promote work–nonwork facilitation.

## Discussion

Job crafting has been shown to be associated with multiple positive work–related outcomes [6–9]. However, whether and how it affects nonwork–related outcomes has been less examined. Based on the COR theory, the current study examined the indirect effect of job crafting on employee general health through work–nonwork facilitation, and the moderating effect of perceived boundary control on this indirect effect. Consistent with COR theory [23, 43], we found that employees who exhibited more job crafting had higher work–nonwork facilitation, and in turn experienced better general health. Further, the indirect effect was stronger for employees with high perceived boundary control than for those with low perceived boundary control. These findings contribute to the limited research on the effect of employees’ job crafting on nonwork–related outcomes, and have practical implications for fostering employee general health.

### Theoretical implications

The findings make several contributions to the literature. First, whereas research to date has mainly focused on the effects of job crafting on work–related outcomes [5, 6, 9], our study extends the research on the relationship between job crafting and non-work outcomes [13, 14, 53]. Following the recent trend to examine the influence of job crafting on employees’ nonwork–related outcomes [13, 14, 53], the present study found a positive effect of job crafting on general health, helping us gain more understanding of the effect of job crafting as it spills over into nonwork life. The results also contribute to the general health literature by showing that in addition to stress (such as personal stress and work stress) [54], bottom–up job designs (e.g., job crafting) are also essential factors to consider as influences on employee general health.

Second, based on COR theory’s [43] concept of work–nonwork facilitation, the present study sheds light on why job crafting can benefit general health. According to this theory, job crafting can be seen as a crucial part of a gain cycle: job crafting might increase resources that lead to further resource gain, thus increasing work–nonwork facilitation and subsequently improving general health. This finding helps explain how employees’ strategies to deal with work demands could be associated with better general health via a resource–related mechanism (improving work–nonwork facilitation).

The current study also enriches the literature on work–nonwork facilitation [34], which was assessed in terms of both work–self facilitation and work–family facilitation. These two forms of work–nonwork facilitation may serve complementary roles as mediators of the relationship between job crafting and general health. However, the form of work–family facilitation has not been tested in previous studies. Work–family facilitation is important to study, given that work and family responsibilities are central to adults’ lives, and adults may prioritize their work and family responsibilities over their health [55]. In addition, the finding that work–nonwork facilitation positively predicted general health not only supports the COR theory, but adds to the literature on the relationship between the work–nonwork interface and general health [56, 57].

This is the first study to examine a boundary condition of the effect of job crafting on general health. The results showed that the effect of job crafting on health through work–nonwork facilitation was stronger for employees who perceived high control over boundaries. The finding suggests a positive interaction between personal resources and job resources. As the level of perceived boundary control increased, employees gained more resources from job crafting, resulting in high work–nonwork facilitation and better general health. This finding is also consistent with the resource gain perspective [58]: the more resources an individual has, the more resources he or she is able to obtain. That is, there is a stacking effect of resources. Those who are able to control the work–family boundary are more likely to benefit from the resources freed up from job crafting, promoting work–nonwork facilitation and ultimately increasing general health.

It has been proposed that the strength of the relationship between environmental resources (e.g., social support, energy) and the work–nonwork interface depends upon individual characteristics such as gender and social class [40]. In support of this proposition, the present study found that the individual characteristic of perceived boundary control increased the relationship between job crafting and work–nonwork facilitation.

### Practical implications

The results have several practical implications for managers and employees. First, the positive relationship between job crafting and general health suggests that interventions or training to increase job crafting could be beneficial for employees and organizations. Prior research found that job crafting interventions were effective in increasing employees’ well–being and health [33, 59]. The steps in one intervention were described by Van den Heuvel et al. [33]. The intervention included a two–day crafting workshop, followed by three or four weekly self–set crafting assignments and a reflection session. Further, managers were encouraged to provide support and freedom for employees’ job crafting to promote better performance in the work and home domains. Employees who could decide how they did their work (job crafting) experienced better general health.

Other potential applied value derives from the finding that work–nonwork facilitation mediated the relationship between job crafting and employee general health. This finding suggests that managers could help employees who combine work and family to realize the benefits of general health. For example, managers could create a work–nonwork supportive organizational culture [60] or provide more developmental opportunities for employees to learn strategies to balance their work and family roles [61]. In addition, employees could promote work–nonwork facilitation by increasing job satisfaction, and social support [62]. For example, individuals may derive pleasure from their positive work experiences by capitalizing on, or savoring, the experiences.

Finally, the results suggest that employees with higher perceived boundary control are more likely to benefit from job crafting’s positive effects on general health than those with lower perceived boundary control. Therefore, employees should increase boundary control, perhaps by communicating their preferred boundary management approach to managers, coworkers, and families. Further, organizations need to be careful to grant additional perceived boundary control to all employees rather than forcing employees to use a particular boundary management strategy. For example, if managers ask employees who prefer combining work and family to increase the separation between these two domains, it may be detrimental to employees’ perceived boundary control.

### Limitations and directions for future research

The present study has several limitations that need to be considered. First, all variables were measured by self-report questionnaires, which may raise concerns about common method variance. Although statistical tests showed that common method variance was not of concern in our data, it will be beneficial in future studies to replicate our findings using data from different sources. Further, although our study used a two-wave design to examine the how job crafting can affect employee general health, any inference of causality among the variables should be made with caution. To allow stronger causal inferences, future studies could use cross–lagged models.

Another limitation is that the moderator we tested was a personal resource (perceived boundary control) as a moderator in the relationship between job crafting and general health. Previous studies have found that work characteristics (e.g., job autonomy, work pressure) moderate the relationship between job crafting and other outcomes [63]. Thus, future studies could explore the moderating role of job characteristics, rather than individual characteristics, in the relationship between job crafting and general health.

Finally, we asked participants to provide the last four digits of their thirteen–digit phone number in order to match the questionnaire data from Time 1 and Time 2. However, there is a possibility that several participants could have the same last four digits. Although we found no duplicates in our data set, a more conservative approach to matching (such as six digits instead of four) should be used in future research.

## Conclusions

The present study suggested that employees who craft their job can potentially promote work–nonwork facilitation, in turn improving general health. In addition, we found that this mediation process was stronger for employees with higher vs. lower perceived boundary control. Together, these results were consistent with the conservation of resources theory and provided support for our hypothesized moderated mediation model. Managers are encouraged to provide opportunities and support for job crafting as a way to promote better nonwork outcomes, including general health.

## Supplementary Information

**Additional file 1.**

## Data Availability

All data generated or analysed during this study are included in this published article and its supplementary information files.
